# Differences in Gene-Gene Interactions in Graves’ Disease Patients Stratified by Age of Onset

**DOI:** 10.1371/journal.pone.0150307

**Published:** 2016-03-04

**Authors:** Beata Jurecka-Lubieniecka, Tomasz Bednarczuk, Rafal Ploski, Jolanta Krajewska, Dorota Kula, Malgorzata Kowalska, Andrzej Tukiendorf, Zofia Kolosza, Barbara Jarzab

**Affiliations:** 1 Department of Nuclear Medicine and Endocrine Oncology, Maria Sklodowska-Curie Memorial Cancer Center and Institute of Oncology, Gliwice Branch, Gliwice, Poland; 2 Department of Internal Medicine and Endocrinology, Medical University of Warsaw, Warsaw, Poland; 3 Department of Medical Genetics, Forensic Medicine, Medical University of Warsaw, Warsaw, Poland; 4 Department of Epidemiology, Maria Sklodowska-Curie Memorial Cancer Center and Institute of Oncology, Gliwice Branch, Gliwice, Poland; IPATIMUP/Faculty of Medicine of the University of Porto, PORTUGAL

## Abstract

**Background:**

Graves’ disease (GD) is a complex disease in which genetic predisposition is modified by environmental factors. Each gene exerts limited effects on the development of autoimmune disease (OR = 1.2–1.5). An epidemiological study revealed that nearly 70% of the risk of developing inherited autoimmunological thyroid diseases (AITD) is the result of gene interactions. In the present study, we analyzed the effects of the interactions of multiple loci on the genetic predisposition to GD. The aim of our analyses was to identify pairs of genes that exhibit a multiplicative interaction effect.

**Material and Methods:**

A total of 709 patients with GD were included in the study. The patients were stratified into more homogeneous groups depending on the age at time of GD onset: younger patients less than 30 years of age and older patients greater than 30 years of age. Association analyses were performed for genes that influence the development of GD: *HLADRB1*, *PTPN22*, *CTLA4* and *TSHR*. The interactions among polymorphisms were analyzed using the multiple logistic regression and multifactor dimensionality reduction (MDR) methods.

**Results:**

GD patients stratified by the age of onset differed in the allele frequencies of the HLADRB1*03 and *1858T* polymorphisms of the *PTPN22* gene (OR = 1.7, p = 0.003; OR = 1.49, p = 0.01, respectively). We evaluated the genetic interactions of four SNPs in a pairwise fashion with regard to disease risk. The coexistence of *HLADRB1* with *CTLA4* or *HLADRB1* with *PTPN22* exhibited interactions on more than additive levels (OR = 3.64, p = 0.002; OR = 4.20, p < 0.001, respectively). These results suggest that interactions between these pairs of genes contribute to the development of GD. MDR analysis confirmed these interactions.

**Conclusion:**

In contrast to a single gene effect, we observed that interactions between the *HLADRB1/PTPN22* and *HLADRB1/CTLA4* genes more closely predicted the risk of GD onset in young patients.

## Introduction

Autoimmune thyroid diseases (AITDs) are multifactorial diseases involving both susceptibility alleles of a number of genes and environmental factors [1,2]. Causal therapy by eliminating autoantigens is not available for the treatment of AITDs [3]. Therefore, the elucidation of the genetic factors that are involved in the pathogenesis of AITDs is critical for early diagnosis and effective treatment [4,5].

Because multiple genes may be involved in the development of GD, investigations of the interactions of multiple genes are essential to understanding the genetic mechanisms underlying this complex disease [6]. Single gene polymorphisms identified in the entire GD population exhibit only a limited effect on the development of autoimmune diseases (OR = 1.2–1.5) [7]. An epidemiological study revealed that nearly 70% of the risk of developing inherited AITD is the result of the interaction of multiple genes [8]. Genetic interactions have been reported in other diseases with immunological backgrounds, such as allergic asthma or rheumatoid arthritis [9,10]. However, in GD, the gene interactions that significantly influence disease development are unknown. The complex interactions among genes and environmental factors complicates the identification of polymorphisms of susceptible genes involved in the development of GD. Young patients are susceptible to environmental factors for a shorter time than older patients, potentially simplifying the identification of a genetic predisposition for the development of complex diseases such as AITDs [11]. Recent studies of the human genome have confirmed the hypothesis that the contribution of genetic predisposition to complex diseases, such as GD, is stronger in young patients. Thus, the age at time of onset is considered one of the most important parameters of a disease phenotype [11]. An association between genetic markers and age at time of onset has been reported in patients with type 1 diabetes, rheumatoid arthritis and multiple sclerosis [12–14].

In the present study, we examined the interactions among genes associated with GD in the Polish population for the first time [15–21]. The patients were divided into two subgroups according to the age criterion of onset (30 years of age at onset of GD), similar to the analyses performed in the whole-genome study.

Our study reveals distinctive features of the genetic predisposition to GD in young patients that include both the participation of single polymorphisms and, in particular, their interactions.

## Materials and Methods

### Subjects

This study consisted of 709 Polish patients who were diagnosed with GD. Patients were recruited between 2003 and 2011 from the Department of Nuclear Medicine and Endocrine Oncology, Center of Oncology, Gliwice, and the Department of Endocrinology, Medical University of Warsaw.

The patients with GD were divided into the following two groups based on age at time of onset of GD: a young patient group with GD onset at ≤ 30 years of age (N = 168); and the older patient group with GD onset at > 30 years of age (N = 541). All patients were of Caucasian origin (S1 Table). The clinical characteristics of the patients are summarized in Table 1. The diagnosis of GD was based on clinical manifestations of hyperthyroidism and at least one of two phenotypes: Graves’ ophthalmopathy or laboratory detection of TSHR autoantibodies (Brahms, Berlin, Germany). The study received approval from the ethics committees of the Medical Research Centre, Polish Academy of Science, and the Maria Sklodowska-Curie Memorial Cancer Centre Institute of Oncology, Gliwice Branch. All GD patients in the investigated cohorts provided informed written consent to participate in the present study.

**Table 1:** General clinical characteristics of patients with GD included in this study.

	N = 709
Gender: female: male; n (%)	562 (79.3): 147 (20.7)
Age of onset in years: valid N; mean ± SD	709; 41.3 ± 14.27
Patients with age of onset ≤ 30 years; valid N, mean age	168; 21.7 ± 6.05
Patients with age of onset > 30 years; valid N, mean age	541; 47.2 ± 10.8
Tobacco smokers: age of onset ≤ 30 years; valid N; n (%)	53 (31.5)
Tobacco smokers: age of onset > 30 years; valid N; n (%)	253 (46.8)

### Methods

Genomic DNA from all participants was extracted from peripheral blood leukocytes using a routine salting-out method. *HLADRB1* genotyping was performed using sequence-specific oligonucleotides (SSOs, Innolipa *HLADRB1*, Innogenetics, Gent, Belgium) and sequence-specific primers (MSSP Class II DRB Only, One Lambda, Dynal All Set SSP DR test, Dynal Biotech, Oslo, Norway) in the Gliwice study, and with a Dynal All Set SSP Dr test (Dynal Biotech, Bromborough, Wirral, UK) in the Warsaw study. The *CTLA4* and *PTPN22* polymorphisms were analyzed using PCF-RFLP methods. In the Gliwice study, PCR was performed using 0.5 units of Hot Star Taq polymerase (Qiagen, Limburg, Germany). PCR products were visualized in 2% agarose gels stained with ethidium bromide and digested with the appropriate restriction enzymes for 3 h at 37°C. The digested DNA fragments were then separated in 3% agarose gels. The identification of the *rs179247* and *rs12101255* polymorphisms in the *TSHR* gene was performed using the TaqMan SNP genotyping Kit (Applied Biosystems, Foster City, CA, USA) according to the manufacturer’s instructions.

## Statistical Analyses

Case-control comparisons of the polymorphisms in different alleles and combinations of the polymorphisms were conducted to estimate the relative risk of developing GD in the two age groups based on odds ratios (with 95% confidence intervals) and the Bonferroni adjustment. The interactions between genes were analyzed based on the multifactor dimensionality reduction (MDR) method, which enabled the detection and characterization of the combined effects of multiple genes in the development of GD in the different age groups. The best model of combined polymorphisms of different loci was selected using tenfold cross-validations and maximum testing accuracy. ORs were calculated using the R package (http://cran.r-project.org). The MDR analysis was performed using MDR software (http://sourceforge.net/projects/mdr/).

## Results

The patients were divided into two groups (a young patient group and an older patient group) according to age at GD onset. To evaluate the genetic interactions in a pairwise fashion, a re-analysis of the allele frequency and genotype distribution of the analyzed polymorphisms was conducted. The allele frequencies of HLADRB1*03 in the *HLADRB1* locus, *1858T* in the *PTPN22* locus, *49G* in the *CTLA4* locus, and *rs179247* in the *TSHR* locus were characterized. The *rs12101255* polymorphism of the *TSRH* gene was not analyzed because of a strong linkage disequilibrium between *rs179247* and *rs12101255* (D’ = 0.99). We evaluated the interactions between these polymorphisms. To minimize the influence of known environmental factors, separate analyses of non-smokers of different ages at time of GD onset were conducted. All four SNPs examined were in Hardy-Weinberg equilibrium.

### Allele frequencies and genotypes distribution

The allele frequencies of the HLADRB1*03 and *1858T* polymorphisms of the *PTPN22* gene differed in GD patients stratified by the age of onset. The frequencies of the HLADRB1*03 allele and 858T allele were significantly higher in the younger patients than in the older patients (30.7% vs. 20.7%, OR = 1.70, p = 0.0031; 22.7% vs. 16.5%, OR = 1.49, p = 0.0187, respectively) (Table 2).

**Table 2:** Frequency of the alleles and distribution of the genotypes of the four genes in patients with GD stratified by age at GD onset.

Gene		Allele/ genotypes	≤30GD,N(%)	>30GD,N (%)	OR (95% CI), ≤30GD vs. > 30D	p* value
PTPN 22	Patients (N)		143	409		
	Allele	C	221 (77.3)	683 (83.5)	1.0 (ref.)	
		T	65 (22.7)	135 (16.5)	1.49 (1.07–2.07)	0.0748
	Genotypes	CC	88 (61.5)	281 (68.7)	1.0 (ref.)	
		CT	45 (31.5)	121 (29.6)	1.19 (0.78–1.80)	1.00
		TT	10 (7.0)	7 (1.7)	4.56 (1.69–12.34)	0.0048
	Carriers	CT+TT	55 (38.5)	128 (31.3)	1.37 (0.92–2.04)	0.4720
CTLA4	Patients (N)		141	480		
	Allele	A	134 (47.5)	487 (50.7)	1.0 (ref.)	
		G	148 (52.5)	473 (49.3)	1.14 (0.87–1.48)	1.00
	Genotypes	AA	36 (25.5)	123 (25.6)	1.0 (ref.)	
		AG	62 (44.0)	241 (50.2)	0.88 (0.55–1.40)	1.00
		GG	43 (30.5)	116 (24.2)	1.27 (0.76–2.11)	1.00
	Carriers	GG+AG	105 (74.5)	357 (74.4)	1.00 (0.65–1.55)	1.00
HLA DRB1	Patients (N)		101	322		
	Allele	DR3-	140 (69.3)	511 (79.3)	1.0 (ref.)	
		DR3+	62 (30.7)	133 (20.7)	1.70 (1.19–2.43)	0.0124
	Genotypes	DR3-/-	42 (41.6)	196 (60.9)	1.0 (ref.)	
		DR3-/+	56 (55.4)	119 (37.0)	2.20 (1.39–3.48)	0.0028
		DR3+/+	3 (3.0)	7 (2.2)	2.00 (0.50–8.05)	1.00
	Carriers	DR3 -/+ or DR3+/+	59 (58.4)	126 (39.1)	2.19 (1.39–3.44)	0.0028
TSHR	Patients (N)		136	386		
	Allele	G	121 (44.5)	383 (49.6)	1.0 (ref.)	
		A	151 (55.5)	389 (50.4)	1.23 (0.93–1.62)	0.5828
	Genotypes	GG	28 (20.6)	110 (28.5)	1.0 (ref.)	
		AG	65 (47.8)	163 (42.2)	1.57 (0.95–2.60)	0.3240
		AA	43 (31.6)	113 (29.3)	1.49 (0.87–2.57)	0.5880
	Carriers	AA+AG	108 (79.4)	276 (71.5)	1.54 (0.96–2.46)	0.2880

*with Bonferroni corrections; GD: Graves’ disease; ≤ 30 GD: patients with age of onset of GD ≤ 30 years; > 30 GD: patients with age of onset of GD > 30 years; OR: odds ratio; 95% CI: 95% confidence interval; OR = 1.0 (ref.): referent category indicating the level of references; Carriers: carriers of minor alleles

Significant differences in the distribution of genotypes were also observed; the frequencies of carriers of the HLADRB1*03 allele and those homozygous for the 1858T allele were significantly higher in younger patients (age-of-onset ≤30 years) than in the older patients (Table 2).

Similar results were obtained for the non-smoking young patients. The frequency of the HLADRB1*03 allele was significantly higher in the young non-smoking patients (N = 65) than in the older non-smoking patients (N = 150) (30.8% vs. 21%, OR = 1.67, p = 0.0293). A higher proportion of young non-smoking patients were carriers of the HLADRB1*03 allele compared to the older non-smoking patients (58.1% vs. 40%, OR = 2.11, p = 0.0128).

The frequency of the *1858T* allele of the PTPN22 gene was significantly higher in the young non-smoking patients (N = 48) than in the older non-smoking patients (N = 58) (25.5% vs. 15.8%, OR = 1.83, p = 0.0055). The genotypes of the *1858T* allele were significantly more diverse in the young non-smoking patients compared to the older non-smoking patients (42.5% vs. 28.8%, OR = 1.83, p = 0.0218).

No significant differences in the frequencies of the 49G polymorphism of the *CTLA4* gene or the *rs179247* polymorphism of the *TSHR* gene were identified between the two age groups. However, a tendency toward a higher frequency of rare alleles of both genes was identified in the young patients compared to the older patients (Table 2).

The risk of GD development in young patients compared to older patients was analyzed by logistic regression. The analysis was performed after adjusting for smoking status, a common environmental risk factor in GD development. Polymorphisms of the PTPN2 and HLADR3 genes were assessed because the genotype distributions of these polymorphisms differed significantly in young and older patients. Young carriers of minor alleles of both HLADRB1*03 and 1858T in PTPN22 polymorphisms exhibited a significantly higher odd ratio (OR) compared to the older carriers (OR = 2.38, p = 0.001, OR = 1.81, p = 0.018, respectively) (Table 3). The results of the logistic regression are consistent with the genotype distribution analyses.

**Table 3:** Odds ratio analysis of young patients compared to older patients with adjustment for smoking. Young/older: number of patients with age of onset of GD ≤ 30 years/number of patients with age of onset of GD > 30 years adjusted by smoking status; carriers: carriers of minor alleles.

		Young/older	OR(95%CI)	*P*
PTPN22	CC	84/260	1 (ref.)	
	CT+TT (carriers)	54/121	1.81 (1.11–2.97)	0.018
HLA DRB1	DR3 -/-	40/181	1 (ref.)	
	DR3 -/+ or DR3+/+ (carriers)	59/120	2.38 (1.47–3.84)	0.001

### Gene-gene interactions effects

The associations of the two-SNP combinations were statistically evaluated by multiple logistic regression. Among the patients with different ages at time of onset, we assessed the differences in the frequencies of the polymorphism pairs of *CTLA4/HLADRB1*, *CTLA4/TSHR*, *CTLA4/PTPN22*, *HLADRB1/TSHR*, *HLADRB1/PTPN22* and *TSHR/PTPN22*. The OR values were estimated compared to the double wild-type genotype.

Significant differences in the frequencies of the HLADRB/CTLA4, HLADRB1/TSHR, HLADRB1/PTPN22, and PTPN22/TSHR pairs of polymorphisms were observed between young and older patients. These pairs of polymorphisms were more prevalent in younger-age-of-onset (≤30) patients than in older patients (41.6% vs. 29.2%; 45.4% vs. 26%; 24.2% vs. 9%; and 32.6% vs. 21.8%, for young patients vs. older patients, respectively).

In young patients, the *PTPN22* polymorphism *1858T*, *CTLA4* polymorphism *49G*, or *TSHR* polymorphism *rs179247* were significantly associated with the disease in a HLADRB1*03 carrier background (OR = 4.2, p = 0.001, OR = 3.64, p = 0.0023, OR = 2.17, p = 0.036, respectively) (Table 4). The coexistence of *HLADRB1* with *CTLA4* and *HLADRB1* with *PTPN22* significantly increased the odds ratio compared to additive effects, suggesting that the interactions between these two genes are involved in the development of GD (OR = 3.64, p = 0.0023 vs. 1.0 × 2.19 = 2.19; OR = 4.20, p = 0.00001 vs. 2.19 × 1.37 = 3.00, respectively) (Table 4). The odds ratio analysis for minor allele carriers was considered in the interaction assessment.

**Table 4:** Differences between the risk of GD onset in young patients compared to older patients according to the coexistence of pairs of polymorphisms. OR: odds ratio; OR = 1.0 (ref.): referent category.

		HLA DR3	
		DRB3-/- (wild type)	DRB3 -/+ and +/+ (carriers)
CTLA4	AA (wild type)	1.0 (ref.)	OR = 4.33 p = 0.003 *
	GG + AG (carriers)	OR = 2.05 p = 0.105	OR = 3.64 p = 0.0023 *
PTPN22	CC (wild type)	1.0 (ref.)	OR = 1.78 p = 0.051 *
	TT + CT (carriers)	OR = 1.18 p = 0.643	OR = 4.2 p = 0.0001 *
TSHR	GG (wild type)	1.0 (ref.)	OR = 1.14 p = 0.771
	AA + AG (carriers)	OR = 0.83 p = 0.622	OR = 2.17 p = 0.036 *

* Statistical significance; carriers: carriers of minor alleles

Pairs of other genes, such as *CTLA4/TSHR* and *CTLA4/PTPN22*, were more frequent in the young patients, resulting in a higher odds ratio in this group. However, this relationship was not statistically significant.

**Fig 1:**
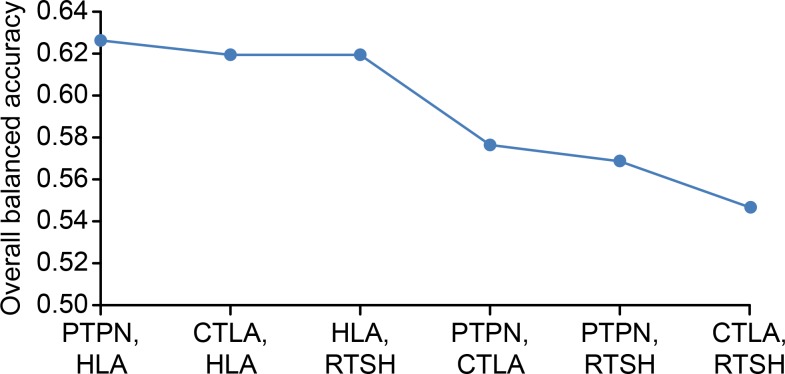
Results of the MDR analysis of the gene pair models differentiating the patient subgroups according to age at time of GD onset.

We re-confirmed interactive genetic effects by MDR analysis to test the 2-way gene-gene interaction model. The MDR method identified 3 pairs of genes, *HLADR3/PTPN22*, *HLADR3/CTLA4*, and *HLADR3/TSHR*, that significantly differentiated young patients from older patients (Fig 1). The SNP HLADRB1*03 had the highest balanced accuracy and cross-validation consistency among the four SNPs, whereas *1858T PTPN22* and HLADRB1*03 had the highest balanced accuracy and best cross-validation consistency among the pairwise interactions (Table 5). *HLA DR3* and *PTPN22* homozygosity for the wild allele were more frequent in older patients, whereas rare allele carriers were more frequent in younger patients.

**Table 5:** Summary of the MDR results.

Best model	Training balanced accuracy	Testing balanced accuracy	Cross-validation consistency
HLADRB1	0.594	0.5835	10/10
PTPN22 –HLADRB1	0.6125	0.5839	9/10

## Discussion

In our study, an association between gene interactions and the risk of GD was identified for the first time in the Polish population using two rounds of analysis. First, subset analysis was conducted to obtain more homogenous research groups. Second, the gene interactions were analyzed using appropriate tests. Complex diseases are heterogeneous; many genes interact with environmental factors and affect the development of disease [22–24]. Heterogeneity hampers the identification of gene polymorphisms that considerably increase the risk of morbidity [25].

Separation of a more homogeneous group of patients according to age at the time of GD onset, including younger-age-of-onset (≤30) patients and older patients, is a simple analytical approach that partially eliminates the limitations associated with the heterogeneity of the analyzed group and shortens the time of the influence of environmental factors.

Our assessment of genetic predisposition to GD in young patients based on the analysis of subgroups of different ages at onset revealed differences in the incidences of the analyzed polymorphisms. At the level of allele frequencies, we identified a relationship between alleles HLADRB1*03 and *1858T* in the gene *PTPN22* and young age at time of onset. Young carriers of HLADRB1*03 or homozygotes of allele *1858T* are more likely to develop GD, despite shorter exposure to environmental factors. The results of the logistic regression are consistent with the genotype distribution analyses. Other polymorphisms, including *CTLA4* and *TSHR*, were more prevalent in younger age-of-onset (≤ 30) patients, but this difference was not significant. The major limitation of this study is the small numbers of patients in both groups, which may complicate the interpretation of negative results.

In the present study we focused on identifying interactions among multiple loci. We performed logistic regression analysis combined with the MDR method to assess gene-gene interactions in the development of GD. The MDR method, which overcomes several limitations of logistic regression, has been widely used for gene-gene interaction analyses [26,27].

The pairs of polymorphisms HLADRB1*03/49G CTLA4, HLADRB1*03/rs179247 TSHR and HLADRB1*03/1858T PTPN22 were closely associated with GD in young patients than in older patients. Patients with a combination of two risk genotypes have an increased risk for GD compared to patients with a single risk genotype. The increase in the odds ratio suggests an interaction of polymorphisms. The HLADRB1*03 and 1858TPTPN22 polymorphisms exhibited an association with GD, both independently and in interactions. Although, the 49G CTLA4 and rs179247TSHR polymorphisms alone did not have significant effects, these polymorphisms do significantly increase disease risk in interactions with other genes.

In the young carriers of the aforementioned genotypes, the risk of GD is higher than that in the elderly patients. The MDR analysis identified the same pairs of polymorphisms.

SNP-based association analyses have become one of the most important approaches for interpreting the underlying molecular mechanisms of complex diseases. We focused on the specific role of polymorphism HLADRB1*03, which was present in every pair of analyzed polymorphisms [28,29]. The molecular mechanisms underlying gene interactions may correspond with the dominant role of HLADRB1*03 in the induction of autoimmune reactions and influence other polymorphisms in the propagation of immune reactions. Subunit A TSHR altered by the intron polymorphism rs179247 is more effectively presented to lymphocytes T by antigen MHC class II HLADRB1*03 [30,31]. The full activation of immune reactions may be propagated because T lymphocyte activation is not hindered due to modifications in the protein molecule products of polymorphism 49G in the *CTLA4* gene and in protein LYP due to the polymorphism *1858T* in the *PTPN22* gene [32,33].

Recent studies have reported only an association between young age at GD onset and single gene polymorphisms. Our previous study of 422 patients confirmed analyses reported by Lavard and Farid [16,34,35]. These studies suggested an association of HLADRB1*03 and young age at time of GD onset in the Caucasian population. Other alleles of *HLADRB1* were identified in young Chinese and Japanese populations [36,37]. In turn, studies by Skorka demonstrated a correlation of *1858T* in gene *PTPN22* with young age at time of GD onset [15].

Interactions between genes *HLA DRB1*, *CTLA4*, *PTPN22* and *TSHR* in the development of GD were not observed in non-age-differentiated groups of GD patients. Based on genetic profiling, Yin et al. did not identify any associations of *HLADRB1* with *CTLA4 49G*, *TSHR rs2268458*, *IL23R rs10889677* or *rs2201841*. Those analyses were conducted in patients without GO in whom GD was diagnosed at an average age of 51 years [38]. Assessing the influence of HLA DRB1*03 with *CTLA4* did not reveal independent effects [39,40]. In the age-differentiated groups of patients with GD, the relationship between PTPN22 and the age at time of GD onset was analyzed. Unfortunately, the gene interaction analysis was conducted for the entire group, and the age at time of onset was not provided. Allele 1858 T of gene *PTPN22*, HLA DRB1*03 and allele *49G* of gene *CTLA4* were identified as independent contributors to the pathogenesis of GD, without significant interactions [15]. We obtained similar results in the present study when comparing the entire group and old patients. The lack of an increase in ORs for coexisting polymorphisms in relation compared to single polymorphisms indicates the independent participation of these polymorphisms in GD pathogenesis in older patients. However, gene interaction effects in the entire group are unclear due to the heterogeneity of this group.

Identifying patient genotypes, including specific pairs of genes, may facilitate early prophylaxis. Additionally, because costimulatory signals are critical for immunological reactions, these molecules may be therapeutic targets for the efficient treatment of patients with GD.

Despite the demonstration of the role of gene-gene interactions in GD development, certain research limitations should be considered. A limitation of this study is the sample size; the separation of groups of patients with specific pairs of polymorphisms decreases the size of these subgroups. Additionally, we could not completely exclude the influence of environmental risks.

To summarize, our study demonstrates that the genetic profiles of young patients with GD differ from those of older patients both in the occurrence of single locus genes and, in particular, the interaction of multiple loci. The gene-gene interactions more closely reflect GD risk than single polymorphisms. The interactions between the *HLADRB1/PTPN22* and *HLADRB1/CTLA4* genes significantly increase the likelihood of GD onset only in young patients.

## Supporting Information

S1 Table GD-patients: age of onset, smoking status, genes. (DOCX)
