# Supplementary material for: Differences in Gene-Gene Interactions in Graves’ Disease Patients Stratified by Age of Onset
Source: PLoS One. 2016 Mar 4;11(3):e0150307. doi: 10.1371/journal.pone.0150307 (PMC4778933; doi:10.1371/journal.pone.0150307)
Supplement: S1 Table — (DOCX) [file pone.0150307.s001.docx]

**Supporting information**

**S1 Tab. GD-patients: age of onset, smoking status, genes.**

| Age of onset (years) | Genes | | | | | Smoking status 0 = non-smoker 1 = smoker |
| --- | --- | --- | --- | --- | --- | --- |
|  | PTPN22 0 = 1 = 2 = | CTLA4 0 = 1 = 2 = | HLADRB1 0 = 1 = 2 = | TSHR rs17 0 = 1 = 2 = | tshrrs12 |  |
| 41,4 | 0 | 2 | 1 | 0 | 0 | 1 |
| 41,9 |  | 1 |  |  |  | 1 |
| 60,5 |  | 2 |  |  |  | 0 |
| 43,6 | 0 | 1 | 0 | 1 | 1 | 1 |
| 59,8 |  | 1 |  |  |  | 0 |
| 40,8 |  | 1 |  |  |  | 0 |
| 50,2 |  | 2 |  |  |  | 0 |
| 37,7 | 1 | 1 | 0 | 0 | 0 | 0 |
| 55,8 | 0 | 1 | 0 | 1 | 1 | 0 |
| 51,3 |  | 2 |  |  |  | 0 |
| 60,5 |  | 2 |  |  |  | 1 |
| 18,3 | 0 | 0 | 1 | 0 | 0 | 0 |
| 52,0 |  | 1 |  |  |  | 0 |
| 45,9 |  | 0 |  |  |  | 1 |
| 41,1 | 0 | 1 | 2 | 0 | 0 | 0 |
| 46,2 |  | 1 |  |  |  | 1 |
| 43,0 |  | 1 |  |  |  | 0 |
| 48,6 |  | 0 |  |  |  | 0 |
| 65,4 | 0 | 1 | 1 | 2 | 2 | 1 |
| 32,9 |  | 0 |  |  |  | 1 |
| 39,5 | 2 | 1 | 1 | 2 | 2 | 0 |
| 30,9 |  | 1 |  |  |  | 0 |
| 67,0 |  | 2 |  |  |  | 0 |
| 52,0 |  | 1 |  |  |  | 1 |
| 32,4 |  | 2 |  |  |  | 0 |
| 39,0 |  | 1 |  |  |  | 1 |
| 48,9 |  | 1 |  |  |  | 1 |
| 46,5 |  | 1 |  |  |  | 1 |
| 55,7 | 0 | 2 | 0 | 2 | 2 | 0 |
| 49,3 | 1 | 1 | 0 | 1 | 1 | 0 |
| 62,0 |  | 0 |  |  |  | 0 |
| 42,4 |  | 0 |  |  |  | 1 |
| 43,2 |  | 1 |  |  |  | 1 |
| 43,4 |  | 1 |  |  |  | 0 |
| 35,6 |  | 2 |  |  |  | 0 |
| 40,2 | 0 | 1 | 1 | 2 | 2 | 0 |
| 44,9 | 1 | 1 | 0 | 1 | 1 | 0 |
| 27,6 |  | 2 |  |  |  | 0 |
| 47,2 |  | 0 |  |  |  | 0 |
| 47,0 | 0 | 0 | 0 | 2 | 1 | 0 |
| 19,0 | 0 | 1 | 1 | 1 | 1 | 1 |
| 14,3 |  | 1 |  |  |  | 1 |
| 49,0 | 0 | 1 | 1 | 0 | 0 | 1 |
| 55,6 |  | 1 |  |  |  | 0 |
| 45,9 |  | 1 |  |  |  | 1 |
| 30,3 |  | 0 |  |  |  | 0 |
| 34,3 |  | 1 |  |  |  | 0 |
| 60,3 | 1 | 1 | 1 | 1 | 1 | 1 |
| 38,7 |  | 1 |  |  |  | 1 |
| 36,7 |  | 2 |  |  |  | 1 |
| 40,2 | 0 | 1 | 2 | 0 | 0 | 0 |
| 60,6 |  | 1 |  |  |  | 0 |
| 26,9 |  | 1 |  |  |  | 0 |
| 38,7 |  | 0 |  |  |  | 0 |
| 54,9 |  | 1 |  |  |  | 0 |
| 30,4 |  | 2 |  |  |  | 1 |
| 43,5 |  | 2 |  |  |  | 1 |
| 48,3 |  | 2 |  |  |  | 1 |
| 52,7 |  | 2 |  |  |  | 1 |
| 41,7 | 0 | 2 | 1 | 0 | 0 | 0 |
| 42,4 | 1 | 1 | 1 | 0 | 0 | 0 |
| 47,9 | 1 | 1 | 0 | 0 | 0 | 1 |
| 23,0 | 0 | 2 | 1 | 0 | 0 | 0 |
| 48,9 |  | 2 |  |  |  | 1 |
| 33,0 |  | 1 |  |  |  | 1 |
| 50,0 |  | 1 |  |  |  | 0 |
| 37,7 |  | 0 |  |  |  | 1 |
| 60,3 |  | 2 |  |  |  | 0 |
| 54,2 | 0 | 2 | 0 | 2 | 1 | 0 |
| 47,3 | 1 | 0 | 0 | 0 | 0 | 1 |
| 50,2 | 0 | 1 | 1 | 2 | 1 | 1 |
| 53,0 | 0 | 1 | 0 | 2 | 2 | 1 |
| 52,8 | 0 | 2 | 0 | 2 | 2 | 0 |
| 57,0 | 1 | 1 | 1 | 1 | 1 | 1 |
| 40,4 |  | 2 |  |  |  | 1 |
| 63,8 | 0 | 1 | 1 | 0 | 0 | 0 |
| 68,3 | 0 | 1 | 1 | 2 | 2 | 1 |
| 40,3 |  | 2 |  |  |  | 0 |
| 47,6 | 1 | 0 | 0 | 1 | 1 | 1 |
| 38,0 |  | 0 |  |  |  | 1 |
| 68,6 | 0 | 1 | 1 | 1 | 1 | 0 |
| 58,5 |  | 0 |  |  |  | 0 |
| 53,6 | 0 | 0 | 1 | 1 | 1 | 0 |
| 48,5 | 0 | 1 | 0 | 2 | 2 | 0 |
| 53,9 |  | 2 |  |  |  | 1 |
| 62,5 | 0 | 1 | 1 | 2 | 1 | 0 |
| 45,3 | 0 | 1 | 1 | 2 | 2 | 1 |
| 52,5 | 1 | 1 | 1 | 1 | 1 | 0 |
| 55,8 | 0 | 2 | 0 | 1 | 1 | 0 |
| 37,3 | 2 | 2 | 0 | 1 | 1 | 0 |
| 46,4 |  | 2 |  |  |  | 0 |
| 30,0 |  | 0 |  |  |  | 0 |
| 47,1 | 1 | 1 | 1 | 2 | 2 | 1 |
| 42,5 |  | 2 |  |  |  | 1 |
| 47,9 | 0 | 1 | 1 | 1 | 1 | 1 |
| 58,4 |  | 1 |  |  |  | 0 |
| 57,3 |  | 0 |  |  |  | 1 |
| 55,4 | 0 | 2 | 0 | 2 | 1 | 0 |
| 51,1 | 0 | 0 | 0 | 2 | 2 | 1 |
| 42,2 |  | 2 |  |  |  | 1 |
| 20,1 |  | 1 |  |  |  | 1 |
| 51,3 | 0 | 1 | 0 | 1 | 1 | 1 |
| 51,1 |  | 1 |  |  |  | 1 |
| 63,4 | 0 | 0 | 0 | 0 | 0 | 0 |
| 39,9 |  | 1 |  |  |  | 1 |
| 67,0 |  | 1 |  |  |  | 1 |
| 39,4 |  | 2 |  |  |  | 1 |
| 64,7 |  | 1 |  |  |  | 1 |
| 31,5 | 0 | 1 | 0 | 0 | 0 | 1 |
| 54,1 | 0 | 1 | 1 | 2 | 2 | 1 |
| 46,4 |  | 1 |  |  |  | 1 |
| 27,0 | 0 | 1 | 0 | 0 | 0 | 1 |
| 24,6 | 0 | 2 | 0 | 2 | 2 | 0 |
| 29,6 |  | 1 |  |  |  | 0 |
| 56,6 | 1 | 0 | 0 | 1 | 0 | 0 |
| 39,5 |  | 2 |  |  |  | 0 |
| 39,2 | 1 | 1 | 1 | 1 | 1 | 1 |
| 44,9 | 0 | 1 | 0 | 2 | 1 | 1 |
| 48,8 |  | 1 |  |  |  | 0 |
| 45,5 |  | 1 |  |  |  | 0 |
| 14,8 |  | 0 |  |  |  | 0 |
| 38,8 | 0 | 0 | 0 | 2 | 2 | 1 |
| 31,3 |  | 1 |  |  |  | 1 |
| 56,0 |  | 0 |  |  |  | 0 |
| 45,2 | 1 | 1 | 1 | 0 | 0 | 0 |
| 68,9 |  | 2 |  |  |  | 0 |
| 45,2 |  | 0 |  |  |  | 1 |
| 58,3 |  | 0 |  |  |  | 0 |
| 60,1 | 0 | 1 | 0 | 2 | 2 | 1 |
| 37,7 |  | 1 |  |  |  | 0 |
| 60,1 |  | 0 |  |  |  | 0 |
| 49,0 | 1 | 1 | 0 | 1 | 1 | 1 |
| 21,1 |  | 0 |  |  |  | 1 |
| 25,6 |  | 1 |  |  |  | 1 |
| 57,6 |  | 2 |  |  |  | 0 |
| 49,8 | 0 | 1 | 0 | 1 | 1 | 0 |
| 80,8 |  | 1 |  |  |  | 0 |
| 48,6 | 1 | 2 | 0 | 0 | 0 | 0 |
| 57,9 | 0 | 0 | 0 | 1 | 1 | 0 |
| 27,4 | 0 | 0 | 0 | 1 | 0 | 0 |
| 39,4 |  | 1 |  |  |  | 0 |
| 57,3 |  | 1 |  |  |  | 1 |
| 34,8 | 1 | 2 | 0 | 1 | 1 | 0 |
| 64,2 |  | 1 |  |  |  | 0 |
| 43,1 | 1 | 2 | 0 | 2 | 1 | 1 |
| 48,7 | 0 | 0 | 0 | 2 | 1 | 1 |
| 53,0 |  | 1 |  |  |  | 0 |
| 54,6 | 0 | 1 | 1 | 0 | 0 | 0 |
| 52,3 | 0 | 1 | 1 | 1 | 1 | 1 |
| 23,3 | 1 | 1 | 0 | 1 | 1 | 0 |
| 60,8 |  | 1 |  |  |  | 1 |
| 20,1 |  | 1 |  |  |  | 1 |
| 61,6 |  | 1 |  |  |  | 0 |
| 30,0 | 0 | 0 | 0 | 2 | 2 | 0 |
| 37,7 | 1 | 0 | 0 | 1 | 1 |  |
| 39,3 | 0 | 1 | 1 | 2 | 0 | 1 |
| 59,6 |  | 2 |  |  |  | 0 |
| 38,9 |  | 1 |  |  |  | 1 |
| 59,1 |  | 2 |  |  |  | 1 |
| 65,1 | 0 | 0 | 0 | 2 | 2 | 0 |
| 52,5 | 0 | 1 | 1 | 1 | 1 | 0 |
| 39,6 |  | 1 |  |  |  | 0 |
| 34,2 | 0 | 2 | 0 | 2 | 1 | 0 |
| 36,7 |  | 1 |  |  |  | 1 |
| 49,5 |  | 1 |  |  |  | 1 |
| 30,4 | 0 | 1 | 0 | 1 | 0 | 1 |
| 53,3 |  | 1 |  |  |  | 1 |
| 36,5 |  | 1 |  |  |  | 0 |
| 30,4 | 1 | 1 | 0 | 1 | 1 | 0 |
| 38,8 | 0 | 2 | 0 | 0 | 0 | 0 |
| 38,5 | 0 | 2 | 0 | 2 | 1 | 1 |
| 44,7 | 1 | 0 | 0 | 2 | 2 | 0 |
| 37,2 | 0 | 0 | 0 | 0 | 0 | 1 |
| 35,1 | 0 | 0 | 1 | 1 | 1 | 0 |
| 45,9 |  | 1 |  |  |  | 0 |
| 27,4 |  | 1 |  |  |  | 0 |
| 40,6 |  | 0 |  |  |  | 1 |
| 28,7 |  | 0 |  |  |  | 0 |
| 59,2 | 1 | 1 | 0 | 0 | 0 | 1 |
| 41,6 |  | 0 |  |  |  | 1 |
| 49,2 | 0 | 1 | 1 | 1 | 1 | 0 |
| 26,4 | 0 | 1 | 1 | 1 | 1 | 0 |
| 15,6 |  | 2 |  |  |  | 0 |
| 45,8 |  | 0 |  |  |  | 0 |
| 41,8 | 0 | 1 | 1 | 1 | 1 | 1 |
| 45,6 |  | 0 |  |  |  | 1 |
| 56,3 |  | 2 |  |  |  | 0 |
| 62,8 | 1 | 0 | 1 | 1 | 0 | 0 |
| 46,7 |  | 1 |  |  |  | 1 |
| 41,8 |  | 1 |  |  |  | 0 |
| 55,0 |  | 1 |  |  |  | 1 |
| 32,1 |  | 0 |  |  |  | 0 |
| 70,8 | 0 | 2 | 0 | 2 | 2 | 0 |
| 44,7 |  | 2 |  |  |  | 0 |
| 34,4 | 0 | 2 | 0 | 2 | 1 | 0 |
| 43,3 |  | 1 |  |  |  | 0 |
| 32,3 | 0 | 1 | 0 | 0 | 0 | 0 |
| 69,9 | 0 | 1 | 0 | 1 | 0 | 0 |
| 50,5 | 0 | 2 | 0 | 0 | 1 | 0 |
| 50,4 | 1 | 2 | 0 | 1 | 0 | 0 |
| 20,4 |  | 2 |  |  |  | 0 |
| 31,4 |  | 2 |  |  |  | 0 |
| 48,7 | 0 | 1 | 0 | 0 | 0 | 0 |
| 53,5 | 0 | 1 | 1 | 0 | 0 | 0 |
| 53,4 |  | 1 |  |  |  | 0 |
| 31,9 |  | 2 |  |  |  | 0 |
| 33,1 |  | 1 |  |  |  | 1 |
| 39,1 |  | 1 |  |  |  | 0 |
| 39,2 |  | 2 |  |  |  | 0 |
| 49,4 | 0 | 2 | 0 | 1 | 0 | 0 |
| 43,7 | 0 | 0 | 1 | 0 | 0 | 0 |
| 26,3 |  | 0 |  |  |  | 0 |
| 28,2 |  | 1 |  |  |  | 1 |
| 34,9 |  | 1 |  |  |  | 0 |
| 35,0 |  | 1 |  |  |  | 0 |
| 42,7 |  | 0 |  |  |  | 0 |
| 37,9 |  | 1 |  |  |  | 0 |
| 43,9 |  | 1 |  |  |  | 0 |
| 39,5 | 0 | 1 | 1 | 2 | 2 | 0 |
| 50,5 | 0 | 2 | 1 | 0 | 0 | 1 |
| 44,8 | 0 | 0 | 0 | 1 | 1 | 1 |
| 36,3 | 0 | 1 | 1 | 0 | 0 | 1 |
| 40,7 | 1 | 1 | 0 | 0 | 0 | 1 |
| 62,4 | 0 | 2 | 0 | 2 | 2 | 1 |
| 28,6 | 1 | 2 | 0 | 1 | 1 | 0 |
| 35,6 | 0 | 1 | 1 | 0 | 0 | 1 |
| 51,8 | 0 | 0 | 0 | 1 | 0 | 1 |
| 30,1 | 0 | 1 | 1 | 0 | 0 | 1 |
| 35,7 | 0 | 0 | 1 | 2 | 1 | 1 |
| 32,8 | 1 | 1 | 1 | 1 | 1 | 1 |
| 63,3 | 2 | 2 | 0 | 0 | 0 | 1 |
| 61,6 | 1 | 0 | 1 | 1 | 0 | 0 |
| 44,0 | 0 | 0 | 1 | 1 | 1 | 0 |
| 29,4 | 0 | 0 | 1 | 2 | 2 | 1 |
| 59,5 | 1 | 2 | 2 | 0 | 0 | 1 |
| 49,7 | 1 | 2 | 1 | 1 | 1 | 1 |
| 26,4 | 1 | 0 | 0 | 0 | 0 | 1 |
| 58,2 | 0 | 1 | 0 | 1 | 1 | 1 |
| 51,3 | 0 | 2 | 1 | 1 | 0 | 0 |
| 55,7 | 0 | 0 | 0 | 2 | 1 | 0 |
| 47,9 | 1 | 2 | 0 | 1 | 1 | 1 |
| 49,1 | 1 | 1 | 0 | 2 | 1 | 0 |
| 16,2 | 0 | 2 | 1 | 0 | 0 | 1 |
| 37,4 | 1 | 1 | 0 | 0 | 0 | 1 |
| 53,5 | 0 | 1 | 0 | 1 | 1 | 1 |
| 50,6 | 0 | 1 | 0 | 1 | 1 | 0 |
| 59,6 | 1 | 1 | 0 | 1 | 1 | 0 |
| 54,8 | 1 | 1 | 1 | 2 | 1 | 1 |
| 45,7 | 1 | 0 | 0 | 1 | 1 | 1 |
| 40,4 | 0 | 1 | 0 | 1 | 1 | 1 |
| 76,5 | 1 | 0 | 0 | 2 | 2 | 1 |
| 58,4 | 0 | 1 | 0 | 1 | 1 | 1 |
| 58,6 | 0 | 2 | 0 | 2 | 1 | 1 |
| 58,5 | 0 | 1 | 1 | 1 | 1 | 0 |
| 16,5 | 0 | 2 | 1 | 1 | 1 | 0 |
| 50,9 | 0 | 0 | 1 | 1 | 1 | 0 |
| 43,8 | 0 | 1 | 2 | 2 | 1 | 0 |
| 54,4 | 1 | 2 | 0 | 2 | 2 | 1 |
| 27,7 | 2 | 1 | 1 | 0 | 0 | 1 |
| 48,3 | 0 | 0 | 1 | 0 | 0 | 1 |
| 19,8 | 1 | 1 | 1 | 2 | 2 | 1 |
| 38,1 | 1 | 0 | 0 |  |  | 1 |
| 14,7 | 0 | 2 | 2 | 1 | 1 | 1 |
| 35,2 | 0 | 1 | 1 | 1 | 1 | 0 |
| 34,0 | 0 | 0 | 0 | 2 | 1 | 1 |
| 61,6 | 0 | 0 | 0 | 1 | 1 | 1 |
| 74,5 | 0 | 0 | 0 | 0 | 0 | 0 |
| 8,4 | 0 | 1 | 1 | 1 | 1 | 1 |
| 47,0 | 0 | 1 | 0 | 0 | 0 | 1 |
| 41,9 | 0 | 0 | 0 | 2 | 2 | 1 |
| 33,2 | 1 | 1 | 0 | 1 | 1 | 0 |
| 70,1 | 1 | 0 | 1 | 1 | 1 | 1 |
| 14,6 | 0 | 2 | 1 | 1 | 0 | 0 |
| 47,0 | 1 | 1 | 0 | 1 | 1 | 1 |
| 30,1 | 1 | 2 | 0 | 2 | 1 | 0 |
| 52,6 | 1 | 0 | 1 | 2 | 0 | 1 |
| 65,8 |  | 1 |  |  |  | 0 |
| 44,9 | 0 | 1 | 0 | 2 | 2 | 0 |
| 32,8 | 0 | 2 | 1 | 1 | 1 | 0 |
| 59,8 |  | 0 |  |  |  | 1 |
| 46,0 | 0 | 1 | 0 | 0 | 0 | 0 |
| 46,4 | 0 | 1 | 0 | 1 | 1 | 1 |
| 52,8 | 1 | 2 | 0 | 1 | 1 | 1 |
| 24,6 | 0 | 1 | 1 | 0 | 0 | 0 |
| 48,8 | 0 | 1 | 1 | 2 | 1 | 0 |
| 41,0 | 0 | 0 | 0 | 2 | 2 | 0 |
| 66,3 |  | 0 |  |  |  | 0 |
| 51,2 | 0 | 1 | 0 | 1 | 1 | 1 |
| 63,2 | 0 | 0 | 0 | 1 | 1 | 0 |
| 22,4 | 0 | 2 | 0 | 0 | 0 | 0 |
| 39,2 | 0 | 0 | 2 | 2 | 2 | 1 |
| 35,2 | 0 | 1 | 1 | 0 | 0 | 0 |
| 34,6 | 1 | 2 | 0 | 2 | 2 | 0 |
| 46,9 |  | 1 |  |  |  | 1 |
| 45,4 | 0 | 1 | 0 | 2 | 2 | 1 |
| 50,8 |  | 1 |  |  |  | 1 |
| 32,8 | 0 | 0 | 0 | 0 | 0 | 1 |
| 18,6 | 1 | 1 | 0 | 1 | 0 | 1 |
| 25,2 | 0 | 0 | 1 | 2 | 0 | 0 |
| 58,0 |  | 0 |  |  |  | 1 |
| 22,7 |  | 1 |  |  |  | 1 |
| 49,8 | 0 | 1 | 0 | 0 | 0 | 1 |
| 46,6 | 1 | 0 | 0 | 1 | 1 | 1 |
| 31,5 | 1 | 1 | 1 | 0 | 0 | 1 |
| 55,0 | 0 | 0 | 0 | 1 | 1 | 1 |
| 44,6 | 0 | 0 | 1 | 1 | 1 | 1 |
| 42,5 | 1 | 1 | 0 | 0 | 0 | 1 |
| 74,0 |  | 1 |  |  |  | 0 |
| 50,7 | 0 | 1 | 0 | 1 | 1 | 1 |
| 59,0 | 0 | 2 | 1 | 0 | 0 | 0 |
| 21,9 | 0 | 1 | 1 | 1 | 1 | 0 |
| 55,1 | 0 | 1 | 1 | 1 | 1 | 1 |
| 40,7 | 0 | 1 | 0 | 0 | 0 | 0 |
| 63,6 |  | 1 |  |  |  | 0 |
| 50,3 | 0 | 1 | 0 | 1 | 1 | 0 |
| 36,2 | 0 | 0 | 1 | 1 | 1 | 0 |
| 26,4 | 1 | 2 | 1 | 1 | 0 | 0 |
| 40,8 | 0 | 1 | 0 | 1 | 1 | 0 |
| 30,4 | 1 | 1 | 0 | 0 | 0 | 0 |
| 56,7 | 1 | 0 | 0 | 0 | 0 | 1 |
| 17,9 | 1 | 2 | 1 | 2 | 2 | 1 |
| 64,6 |  | 2 |  |  |  | 0 |
| 23,4 | 0 | 2 | 0 | 1 | 1 | 1 |
| 46,8 | 0 | 1 | 0 | 1 | 1 | 0 |
| 54,0 | 1 | 2 | 0 | 2 | 1 | 0 |
| 30,5 | 0 | 1 | 1 |  |  | 1 |
| 33,6 | 0 | 1 | 0 | 1 | 1 | 0 |
| 30,0 | 0 | 1 | 1 | 2 | 1 | 0 |
| 72,9 |  | 1 |  |  |  | 0 |
| 38,6 | 1 | 0 | 1 | 0 | 0 | 1 |
| 30,4 | 0 | 1 | 1 | 1 | 1 | 1 |
| 53,9 | 0 | 1 | 0 | 1 | 1 | 1 |
| 20,6 | 1 | 2 | 1 | 0 | 0 | 0 |
| 46,0 | 0 | 1 | 1 | 2 | 0 | 1 |
| 48,2 |  |  |  |  |  | 1 |
| 65,4 |  |  |  |  |  | 1 |
| 45,7 |  |  |  |  |  | 0 |
| 46,0 | 1 | 1 | 2 | 1 | 1 |  |
| 22,0 | 1 | 1 | 1 |  |  | 0 |
| 22,0 | 0 | 1 | 1 | 2 | 2 | 0 |
| 21,0 | 0 | 1 | 1 | 0 | 0 | 0 |
| 45,0 | 0 | 0 | 1 | 1 | 1 | 1 |
| 14,0 | 1 | 0 | 1 | 1 | 0 | 0 |
| 67,0 | 1 | 2 | 0 | 2 | 1 |  |
| 25,0 | 0 | 1 | 1 | 1 | 0 | 1 |
| 27,0 | 2 | 2 | 1 | 1 | 1 | 1 |
| 47,0 | 0 | 2 | 0 | 1 | 0 | 1 |
| 22,8 | 1 | 0 | 1 | 1 | 1 | 0 |
| 17,0 | 0 | 0 | 1 | 2 | 1 | 0 |
| 32,5 | 1 | 2 | 1 | 1 | 1 | 1 |
| 56,0 | 0 | 2 | 1 | 1 | 1 | 0 |
| 57,0 | 0 | 1 | 1 | 2 | 2 |  |
| 63,0 | 0 | 2 | 0 | 0 | 0 | 1 |
| 17,0 | 2 | 0 | 0 | 0 | 0 | 0 |
| 28,0 | 0 | 2 | 0 |  |  | 0 |
| 51,0 | 0 | 0 | 0 | 1 | 1 |  |
| 41,0 | 0 | 1 | 0 | 2 | 1 |  |
| 48,0 | 0 | 1 | 0 |  |  | 0 |
| 50,0 | 0 | 1 | 0 | 1 | 0 | 0 |
| 47,0 | 0 | 1 | 0 | 2 | 2 | 1 |
| 32,0 | 1 | 1 | 0 | 2 | 2 | 0 |
| 19,0 | 0 | 2 | 1 | 0 | 0 | 1 |
| 55,0 | 1 | 1 | 0 | 2 | 2 | 0 |
| 26,0 | 0 | 1 | 0 | 1 | 1 |  |
| 21,0 | 0 | 0 |  | 1 | 1 | 0 |
| 35,0 | 0 | 0 |  | 2 | 1 | 0 |
| 3,0 | 0 | 1 |  | 1 | 1 | 0 |
| 47,0 | 0 | 1 |  | 0 | 0 | 1 |
| 35,0 | 0 | 1 |  |  |  | 1 |
| 12,0 | 0 | 2 |  | 0 | 0 | 0 |
| 34,5 | 0 | 2 |  | 2 | 2 | 0 |
| 14,0 | 1 | 2 |  | 1 | 0 | 0 |
| 18,0 | 0 | 2 |  | 2 | 2 | 0 |
| 15,0 | 1 | 2 |  | 2 | 1 | 0 |
| 21,0 | 1 |  |  | 1 | 1 | 0 |
| 41,0 | 0 |  |  | 0 | 0 | 1 |
| 32,0 | 1 |  |  | 1 | 0 | 0 |
| 41,3 | 0 |  |  |  |  | 1 |
| 23,0 | 0 |  |  | 1 | 0 | 0 |
| 27,0 | 1 | 1 | 0 | 1 | 1 | 0 |
| 46,5 | 1 | 0 | 0 | 2 | 2 | 1 |
| 41,9 | 1 | 0 | 0 | 2 | 2 | 1 |
| 42,0 | 0 | 1 | 1 | 0 | 0 | 1 |
| 46,0 | 0 | 2 | 0 | 2 | 2 | 0 |
| 28,9 | 1 | 0 | 1 | 1 | 1 | 0 |
| 30,0 | 0 | 1 | 0 | 1 | 1 | 0 |
| 30,9 | 1 | 0 | 0 | 1 | 0 | 0 |
| 43,0 | 0 | 0 | 0 | 0 | 0 | 0 |
| 61,0 | 0 | 1 | 0 |  |  | 1 |
| 52,0 | 0 | 1 | 0 | 1 | 1 | 1 |
| 35,0 | 1 | 1 | 0 | 1 | 1 | 1 |
| 45,0 | 0 | 2 | 0 | 1 | 1 | 1 |
| 17,0 | 0 | 0 | 1 | 0 | 0 | 0 |
| 41,0 | 0 | 0 |  | 2 | 2 | 0 |
| 61,0 | 1 | 1 |  | 0 | 0 | 1 |
| 67,0 | 1 | 2 |  | 2 | 1 | 0 |
| 48,0 | 0 |  |  |  |  | 1 |
| 29,0 | 0 |  |  | 1 | 1 | 1 |
| 52,0 | 0 | 2 | 0 | 1 | 1 | 1 |
| 40,0 | 1 | 2 | 0 | 1 | 0 | 0 |
| 35,0 | 1 | 1 | 0 | 2 | 2 | 1 |
| 53,0 | 0 | 1 | 0 | 1 | 1 | 1 |
| 53,0 | 0 | 0 | 0 | 1 | 0 | 1 |
| 43,0 | 0 | 0 | 0 | 1 | 1 | 0 |
| 47,0 | 0 |  |  | 2 | 1 | 1 |
| 61,0 | 1 |  |  | 0 | 0 | 1 |
| 24,0 | 0 | 2 | 1 | 1 | 1 | 0 |
| 12,0 |  |  |  | 2 | 2 | 0 |
| 47,0 | 1 |  |  |  |  | 0 |
| 32,0 | 0 |  |  | 2 | 1 |  |
| 29,0 | 1 |  |  | 1 | 1 | 0 |
| 48,0 | 1 |  |  | 0 | 0 | 0 |
| 72,0 | 0 |  |  |  |  | 1 |
| 22,0 | 0 |  |  | 2 | 2 | 0 |
| 58,0 | 0 |  |  | 2 | 2 | 1 |
| 40,8 |  |  |  | 2 | 2 |  |
| 18,0 | 1 | 0 | 1 | 1 | 1 | 0 |
| 55,0 | 0 | 1 | 1 | 0 | 0 | 0 |
| 61,0 | 0 | 1 | 2 | 0 | 0 |  |
| 53,0 | 0 | 1 | 1 | 2 | 0 | 0 |
| 47,5 | 0 | 2 | 1 | 1 | 0 | 0 |
| 43,0 | 0 | 1 | 1 | 2 | 2 | 0 |
| 42,0 | 0 | 0 | 1 | 1 | 1 | 0 |
| 25,0 | 0 | 0 | 1 | 0 | 0 | 1 |
| 42,0 | 0 | 1 | 1 | 2 | 2 |  |
| 55,0 | 0 | 1 | 1 | 2 | 2 | 0 |
| 18,0 | 1 | 1 | 1 | 1 | 1 | 0 |
| 28,0 | 0 | 1 | 1 |  |  | 1 |
| 20,0 | 1 | 2 | 1 | 1 | 1 | 1 |
| 35,0 | 0 | 2 | 0 | 1 | 0 | 1 |
| 21,0 | 0 | 1 | 1 | 2 | 1 | 0 |
| 60,0 | 1 | 1 | 1 | 0 | 0 | 1 |
| 23,0 | 0 | 1 | 1 | 1 | 0 | 1 |
| 38,0 | 0 | 1 | 1 | 1 | 0 | 0 |
| 41,0 | 1 | 2 | 1 | 0 | 0 | 1 |
| 43,0 | 0 | 2 | 1 | 2 | 2 | 1 |
| 37,0 | 0 | 1 | 0 | 0 | 0 |  |
| 16,0 | 0 | 1 | 0 | 2 | 1 | 0 |
| 40,0 | 1 | 1 | 0 | 2 | 1 | 1 |
| 49,0 | 0 | 0 | 1 | 0 | 0 | 1 |
| 43,0 | 0 | 2 | 1 | 0 | 0 | 0 |
| 57,0 | 0 | 2 | 1 | 0 | 0 | 0 |
| 37,3 | 0 | 0 | 0 | 0 | 0 | 1 |
| 34,0 | 1 | 0 | 0 | 0 | 1 | 0 |
| 37,0 | 0 | 0 | 1 | 2 | 2 | 0 |
| 32,0 | 0 | 2 | 0 | 1 | 0 | 0 |
| 20,0 | 0 | 1 | 0 | 0 | 0 | 0 |
| 33,0 | 0 | 1 | 0 | 1 | 1 | 0 |
| 24,0 | 0 | 1 | 0 | 1 | 1 |  |
| 45,0 | 0 | 2 | 0 | 0 | 0 |  |
| 49,0 | 0 | 2 | 0 | 2 | 2 | 0 |
| 18,0 | 0 | 1 | 0 | 2 | 2 | 0 |
| 45,0 | 0 | 1 | 0 | 1 | 1 | 0 |
| 36,0 | 0 | 2 | 0 | 1 | 1 | 1 |
| 31,0 | 1 | 2 | 0 |  |  | 0 |
| 67,0 | 1 | 2 | 0 | 2 | 1 | 0 |
| 59,0 | 0 | 1 | 0 |  |  | 0 |
| 49,0 | 1 | 1 | 0 | 2 | 2 |  |
| 37,0 | 0 | 1 | 0 | 0 | 0 |  |
| 37,0 | 0 | 0 | 1 | 0 | 0 | 1 |
| 15,0 | 0 | 1 | 0 | 1 | 1 | 0 |
| 36,0 | 0 | 1 | 0 | 1 | 0 | 0 |
| 52,0 | 0 | 0 | 0 | 0 | 0 | 1 |
| 37,0 | 1 | 0 | 0 |  |  | 1 |
| 46,0 | 1 | 1 | 0 | 2 | 1 | 0 |
| 21,0 | 1 | 1 | 0 | 0 | 0 | 1 |
| 30,0 | 2 | 1 | 0 | 1 | 1 | 0 |
| 29,5 | 0 | 0 | 0 | 1 | 1 | 0 |
| 22,0 | 0 | 0 | 1 | 1 | 1 | 0 |
| 23,5 | 1 | 1 | 0 | 2 | 2 | 0 |
| 47,0 | 0 | 1 | 0 | 0 | 0 | 1 |
| 45,0 | 0 | 2 | 0 | 2 | 2 | 1 |
| 37,0 | 1 | 2 | 0 | 2 | 2 |  |
| 37,0 | 2 | 1 | 0 | 2 | 2 | 0 |
| 52,0 | 0 | 0 | 0 | 1 | 1 | 0 |
| 61,0 | 0 | 0 | 0 |  |  | 0 |
| 19,0 | 1 | 0 |  | 1 | 1 | 0 |
| 9,0 | 2 | 0 |  | 1 | 0 | 0 |
| 18,5 | 1 | 0 |  | 2 | 1 | 0 |
| 35,0 | 1 | 0 |  | 1 | 0 | 1 |
| 60,0 | 0 | 1 |  | 2 | 2 | 0 |
| 43,0 | 1 | 1 |  | 2 | 1 | 0 |
| 46,0 | 1 | 1 |  | 1 | 0 | 1 |
| 36,0 | 1 | 1 |  | 1 | 1 | 1 |
| 43,0 | 1 | 1 |  | 1 | 1 | 0 |
| 31,0 | 0 | 1 |  | 1 | 1 |  |
| 51,0 | 1 | 2 |  | 1 | 0 | 1 |
| 14,0 | 1 | 2 |  | 1 | 0 | 0 |
| 34,0 | 1 | 2 |  | 2 | 0 | 1 |
| 50,0 | 0 | 2 |  | 1 | 1 |  |
| 6,0 | 1 | 2 |  | 2 | 2 | 0 |
| 25,0 | 1 |  |  | 1 | 0 | 1 |
| 24,0 | 0 |  |  |  |  | 1 |
| 51,0 | 0 |  |  | 0 | 0 | 1 |
| 17,0 | 0 |  |  | 1 | 1 | 1 |
| 51,0 | 0 |  |  | 0 | 0 | 0 |
| 37,0 | 0 |  |  | 0 | 0 | 1 |
| 64,0 | 2 |  |  |  |  | 0 |
| 49,0 | 0 |  |  | 2 | 1 | 1 |
| 51,0 | 0 |  |  | 1 | 0 | 1 |
| 52,0 | 0 |  |  | 1 | 1 | 1 |
| 45,0 | 1 |  |  | 1 | 1 | 1 |
| 51,0 | 1 |  |  |  |  | 1 |
| 27,0 | 0 |  |  | 0 | 1 | 0 |
| 28,0 | 0 |  |  |  |  | 0 |
| 47,0 | 0 |  |  |  |  | 1 |
| 38,0 | 1 |  |  | 1 | 1 | 0 |
| 20,0 | 2 | 0 | 1 | 0 | 0 | 0 |
| 28,0 | 0 | 2 | 1 | 0 | 0 | 0 |
| 41,0 | 0 | 2 | 1 | 0 | 0 | 0 |
| 55,0 | 0 | 0 | 1 | 1 | 0 | 1 |
| 51,9 | 0 | 0 | 1 | 0 | 0 | 1 |
| 35,0 | 0 | 0 | 1 | 1 | 1 | 1 |
| 20,0 | 1 | 1 | 1 | 1 | 1 | 0 |
| 49,0 | 0 | 1 | 1 | 1 | 1 | 0 |
| 23,0 | 1 | 1 | 1 | 2 | 2 | 0 |
| 47,0 | 0 | 2 | 1 | 1 | 1 |  |
| 44,0 | 0 | 0 | 0 | 1 | 1 | 1 |
| 45,0 | 0 | 2 | 1 | 0 | 0 | 0 |
| 32,0 | 0 | 1 | 1 | 2 | 2 | 1 |
| 13,0 | 0 | 1 | 1 | 2 | 1 | 0 |
| 22,0 | 0 | 2 | 1 | 1 | 1 | 1 |
| 29,0 | 0 | 1 | 1 | 1 | 0 | 0 |
| 43,0 | 0 | 2 | 1 | 0 | 0 | 0 |
| 27,0 | 0 | 1 | 0 | 0 | 0 | 1 |
| 23,0 | 0 | 2 | 1 | 2 | 0 | 1 |
| 47,0 | 1 | 0 | 0 | 0 | 0 | 0 |
| 32,0 | 1 | 1 | 0 | 0 | 0 |  |
| 50,0 | 0 | 0 | 1 | 2 | 2 | 0 |
| 42,5 | 0 | 0 | 0 | 2 | 1 | 1 |
| 49,0 | 0 | 1 | 0 | 1 | 1 | 0 |
| 18,0 | 1 | 0 | 1 | 2 | 2 | 0 |
| 43,0 | 0 | 0 | 0 | 1 | 1 |  |
| 45,0 | 1 | 1 | 0 | 1 | 1 | 0 |
| 17,0 | 2 | 2 | 0 | 2 | 2 | 0 |
| 21,0 | 0 | 2 | 0 | 1 | 1 | 1 |
| 23,0 | 1 | 1 | 0 | 0 | 0 | 0 |
| 28,5 | 0 | 0 | 0 | 1 | 1 | 0 |
| 37,0 | 1 | 2 | 0 | 0 | 0 | 1 |
| 21,0 | 2 | 1 | 1 | 1 | 1 | 0 |
| 47,0 | 0 | 1 | 0 | 0 | 0 | 1 |
| 47,0 | 0 | 1 | 0 | 0 | 0 | 1 |
| 38,0 | 1 | 1 | 0 | 2 | 1 | 0 |
| 20,0 | 0 | 2 | 0 | 1 | 0 | 1 |
| 34,0 | 0 | 1 | 0 | 1 | 1 | 0 |
| 44,5 | 1 | 0 | 0 | 1 | 0 | 1 |
| 22,0 |  | 2 | 1 |  |  | 1 |
| 53,0 | 0 | 0 |  | 1 | 1 | 1 |
| 42,8 | 1 | 0 |  | 1 | 1 | 1 |
| 6,0 | 0 | 1 |  | 1 | 1 | 0 |
| 42,0 | 1 | 1 |  | 0 | 0 | 0 |
| 50,0 | 0 | 1 |  | 1 | 1 | 1 |
| 55,0 | 0 | 1 |  | 0 | 0 | 0 |
| 5,0 | 1 | 2 |  | 1 | 1 | 0 |
| 14,0 | 0 | 2 |  | 1 | 0 | 0 |
| 44,0 | 0 |  |  |  |  | 1 |
| 19,5 | 0 |  |  | 0 | 0 | 1 |
| 34,0 | 0 |  |  | 1 | 1 | 0 |
| 67,0 |  |  |  |  |  |  |
| 28,0 | 0 |  |  | 2 | 1 | 1 |
| 15,0 | 0 |  |  |  |  |  |
| 19,0 | 1 | 1 | 1 | 2 | 2 | 0 |
| 38,0 | 1 | 1 | 1 | 0 | 0 | 1 |
| 26,0 | 2 | 1 | 0 | 1 | 0 | 0 |
| 45,5 | 0 | 0 | 0 | 0 | 0 | 0 |
| 40,5 | 0 | 0 | 0 | 1 | 0 | 1 |
| 48,9 | 0 | 0 | 1 | 1 | 0 | 1 |
| 47,0 | 0 |  |  | 2 | 1 |  |
| 43,0 | 0 |  |  |  |  | 1 |
| 47,0 | 0 | 0 | 0 | 1 | 1 | 0 |
| 28,0 |  | 1 | 0 | 2 | 0 | 0 |
| 51,0 |  |  |  | 1 | 1 | 1 |
| 26,0 | 1 |  |  |  |  |  |
| 34,0 | 0 |  |  | 1 | 1 | 0 |
| 16,0 | 0 |  |  |  |  | 0 |
| 34,0 | 0 |  |  |  |  | 0 |
| 73,0 |  |  |  | 0 | 0 | 0 |
| 23,0 | 0 |  |  | 2 | 0 | 0 |
| 19,0 | 0 |  |  |  |  | 1 |
| 48,0 | 0 |  |  | 2 | 2 | 0 |
| 35,0 | 0 |  |  |  |  | 0 |
| 50,0 | 0 |  |  |  |  | 0 |
| 49,0 | 0 |  |  | 1 | 1 | 0 |
| 64,0 | 0 |  |  | 0 | 0 | 1 |
| 29,0 |  |  |  | 2 | 1 |  |
| 45,0 |  |  |  |  |  | 0 |
| 26,0 |  |  |  | 2 | 2 |  |
| 26,0 | 1 | 1 | 1 | 1 | 1 | 1 |
| 49,0 | 0 | 2 | 1 | 2 | 2 | 0 |
| 58,0 | 0 | 0 | 0 | 1 | 1 | 0 |
| 41,9 | 0 | 0 | 1 | 2 | 2 | 1 |
| 53,0 | 0 | 2 | 1 | 1 | 0 | 0 |
| 19,0 | 2 | 0 | 1 | 1 | 1 | 0 |
| 30,9 | 1 | 0 | 1 |  |  | 0 |
| 38,0 | 0 | 2 | 1 | 2 | 2 | 0 |
| 48,0 | 0 | 0 | 1 | 0 | 0 |  |
| 41,0 | 0 | 1 | 1 | 1 | 1 | 0 |
| 48,0 | 0 | 1 | 1 | 0 | 1 | 1 |
| 31,0 | 0 | 2 | 1 | 1 | 1 | 1 |
| 44,0 | 0 | 2 | 1 | 1 | 0 | 0 |
| 62,0 | 0 | 2 | 0 | 0 | 0 | 1 |
| 13,0 | 1 | 1 | 1 | 1 | 0 | 0 |
| 70,0 | 0 | 0 | 1 | 1 | 0 | 0 |
| 47,0 | 1 | 2 | 1 | 1 | 0 | 1 |
| 27,0 | 0 | 2 | 0 | 1 | 1 | 1 |
| 31,0 | 0 | 1 |  | 2 | 1 | 0 |
| 43,0 | 1 | 0 | 1 | 2 | 2 | 1 |
| 36,0 | 0 | 1 | 1 | 1 | 1 | 0 |
| 35,0 | 1 | 1 | 0 | 2 | 1 | 1 |
| 57,0 | 1 | 1 | 0 | 1 | 1 | 1 |
| 41,5 | 0 | 1 | 1 |  |  | 1 |
| 38,0 | 0 | 0 | 0 | 0 | 0 |  |
| 42,0 | 0 | 1 | 0 | 1 | 1 | 1 |
| 29,5 | 0 | 0 | 0 | 1 | 1 | 1 |
| 57,0 | 0 | 1 | 0 | 1 | 0 | 1 |
| 30,0 | 1 | 2 | 0 | 0 | 0 | 0 |
| 44,0 | 0 | 1 | 0 | 0 | 0 | 0 |
| 28,0 | 0 | 0 | 0 | 2 | 1 | 0 |
| 53,0 | 1 | 1 | 0 | 1 | 1 |  |
| 21,0 | 0 | 1 | 0 | 0 | 0 | 0 |
| 47,0 | 0 | 1 | 0 | 1 | 1 | 0 |
| 47,0 | 0 | 0 | 1 | 1 | 1 | 1 |
| 43,0 | 1 | 0 | 0 | 1 | 1 | 1 |
| 45,0 | 0 | 1 | 0 | 2 | 2 | 1 |
| 41,5 | 0 | 2 | 0 | 0 | 0 | 0 |
| 77,0 | 0 | 1 | 0 | 1 | 1 |  |
| 45,0 | 0 | 0 | 0 | 0 | 0 | 1 |
| 51,0 | 0 | 1 | 0 | 0 | 0 | 0 |
| 16,0 | 0 | 1 | 0 | 0 | 0 | 1 |
| 43,0 | 1 | 0 |  | 0 | 0 | 1 |
| 34,0 | 0 | 0 |  | 1 | 1 | 1 |
| 22,0 | 0 | 0 |  | 1 | 1 | 0 |
| 40,0 | 1 | 1 |  | 0 | 0 | 1 |
| 53,0 | 1 | 1 |  |  |  | 0 |
| 48,0 | 1 | 1 |  | 0 | 0 | 1 |
| 43,0 | 0 | 1 |  |  |  | 1 |
| 34,0 | 1 | 1 |  | 1 | 1 | 0 |
| 28,0 | 1 | 2 |  | 2 | 1 | 1 |
| 5,0 | 0 | 2 |  | 0 | 0 | 0 |
| 7,0 | 0 | 2 |  | 1 | 1 | 0 |
| 42,0 | 0 | 2 |  | 1 | 1 | 1 |
| 30,0 | 0 |  |  | 2 | 2 | 1 |
| 45,0 | 2 |  |  |  |  | 0 |
| 58,0 | 0 |  |  | 2 | 2 | 1 |
| 53,0 | 0 |  |  | 2 | 0 | 0 |
| 22,0 | 0 | 0 | 2 | 1 | 1 | 0 |
| 51,0 | 0 | 1 | 1 | 1 | 1 | 1 |
| 41,0 | 1 | 0 | 1 | 0 | 0 | 0 |
| 45,0 | 1 | 2 | 1 | 0 | 0 | 0 |
| 69,0 | 0 | 1 | 1 | 1 | 0 | 0 |
| 28,0 | 1 | 1 | 0 | 2 | 0 | 0 |
| 28,0 | 0 | 2 | 0 | 2 | 1 | 1 |
| 24,9 | 1 | 0 | 1 | 2 | 2 | 1 |
| 65,5 | 0 | 1 | 1 | 0 | 0 | 1 |
| 46,0 | 0 | 1 | 1 | 1 | 1 | 1 |
| 45,0 | 0 | 1 | 0 | 0 | 0 | 1 |
| 14,0 | 0 | 1 | 1 | 2 | 1 | 0 |
| 29,0 | 0 | 2 | 1 | 1 | 0 | 1 |
| 38,0 | 0 | 1 | 0 | 1 | 0 | 0 |
| 42,0 | 0 | 0 | 0 | 1 | 1 | 1 |
| 56,0 | 1 | 1 | 0 | 2 | 2 | 1 |
| 48,0 | 0 | 1 | 0 |  |  | 0 |
| 69,5 | 1 | 0 | 0 | 1 | 1 | 0 |
| 35,0 | 0 | 1 | 1 | 0 | 0 | 0 |
| 15,0 | 1 | 0 | 2 | 2 | 2 | 0 |
| 50,0 | 1 | 0 |  | 0 | 0 | 1 |
| 64,0 |  | 1 |  |  |  | 0 |
| 30,0 | 0 | 2 |  |  |  | 0 |
| 50,0 | 0 | 2 |  | 2 | 1 |  |
| 35,0 | 0 | 2 |  | 1 | 1 | 0 |
| 48,0 | 1 |  |  | 0 | 0 | 0 |
| 26,0 | 1 |  |  | 2 | 2 | 1 |
| 40,0 | 1 | 2 | 0 | 0 | 0 | 1 |
| 40,0 | 1 | 2 | 1 | 0 | 0 | 1 |
| 27,0 | 1 | 1 | 0 | 0 | 1 | 1 |
| 31,0 | 0 | 1 | 0 | 0 | 0 | 0 |
| 20,0 | 0 | 0 |  | 1 | 1 | 1 |
| 50,0 | 2 | 1 |  | 1 | 0 | 1 |
| 25,0 | 0 | 1 | 0 | 1 | 0 | 0 |
| 24,0 | 0 | 1 |  | 2 | 2 | 0 |
| 14,0 | 0 | 1 |  | 2 | 1 | 0 |
| 43,0 | 1 |  |  | 1 | 0 | 1 |
| 38,0 | 0 |  |  | 2 | 1 |  |
| 51,0 | 0 |  |  | 0 | 0 | 1 |
| 41,0 | 0 |  |  | 2 | 1 |  |
| 14,0 | 0 |  |  | 2 | 2 | 0 |
| 42,0 | 0 |  |  | 1 | 1 | 1 |
| 57,0 | 0 |  |  | 1 | 1 | 1 |
| 33,0 | 0 |  |  | 1 | 1 | 0 |
| 47,0 | 1 |  |  | 2 | 1 | 1 |
| 22,0 | 1 |  |  |  |  | 0 |
| 55,0 | 0 |  |  | 0 | 0 | 0 |
| 70,0 | 0 |  |  | 2 | 2 | 0 |
| 58,0 | 0 |  |  |  |  | 0 |
| 40,0 | 0 |  |  |  |  | 0 |
| 42,5 |  |  |  | 2 | 1 |  |
| 40,0 | 0 | 1 | 0 | 1 | 0 |  |
| 35,0 | 1 | 2 | 0 | 2 | 1 | 0 |
| 33,0 | 0 | 2 |  | 1 | 1 | 1 |
| 30,0 | 0 | 0 |  | 2 | 1 |  |
| 59,0 | 0 | 2 |  | 0 | 0 | 1 |
| 26,0 | 1 | 1 | 0 | 2 | 1 | 0 |
| 43,0 | 0 | 0 |  | 2 | 2 | 1 |
| 24,0 |  |  |  |  |  | 0 |
| 55,0 |  |  |  | 2 | 2 | 0 |
| 26,0 |  |  |  |  |  | 1 |
| 12,0 |  |  |  |  |  | 0 |
| 41,0 |  |  |  |  |  | 1 |
| 47,0 |  |  |  | 1 | 1 | 0 |

0 = homozygosity for the wild allele

1 = heterozygosity for the wild and risk allele

2 = homozygosity for the risk allele
